# A Solar Panel-Integrated Modified Planner Inverted F Antenna for Low Earth Orbit Remote Sensing Nanosatellite Communication System

**DOI:** 10.3390/s18082480

**Published:** 2018-07-31

**Authors:** Touhidul Alam, Mohammad Tariqul Islam, Md. Amanath Ullah, Mengu Cho

**Affiliations:** 1 Centre of Advanced Electronic and Communication Engineering, Faculty of Engineering and Built Environment, Universiti Kebangsaan Malaysia, Bangi, Selangor 43600, Malaysia; touhid13@siswa.ukm.edu.my (T.A.); amanath@siswa.ukm.edu.my (M.A.U.); 2 Laboratory of Spacecraft Environment Interaction Engineering (LaSEINE), Kyushu Institute of Technology, Kitakyushu 804-8550, Japan; cho@ele.kyutech.ac.jp

**Keywords:** remote sensing CubeSat, deployment free, nanosatellite, modified PIFA, UHF antenna, UHF communication

## Abstract

One of the most efficient methods to observe the impact of geographical, environmental, and geological changes is remote sensing. Nowadays, nanosatellites are being used to observe climate change using remote sensing technology. Communication between a remote sensing nanosatellite and Earth significantly depends upon antenna systems. Body-mounted solar panels are the main source of satellite operating power unless deployable solar panels are used. Lower ultra-high frequency (UHF) nanosatellite antenna design is a crucial challenge due to the physical size constraint and the need for solar panel integration. Moreover, nanosatellite space missions are vulnerable because of antenna and solar panel deployment complexity. This paper proposes a solar panel-integrated modified planner inverted F antenna (PIFA) to mitigate these crucial limitations. The antenna consists of a slotted rectangular radiating patch with coaxial probe feeding and a rectangular ground plane. The proposed antenna has achieved a −10 dB impedance bandwidth of 6.0 MHz (447.5 MHz–453.5 MHz) with a small-sized (80 mm× 90 mm× 0.5 mm) radiating element. In addition, the antenna achieved a maximum realized gain of 0.6 dB and a total efficiency of 67.45% with the nanosatellite structure and a solar panel. The challenges addressed by the proposed antenna are to ensure solar panel placement between the radiating element and the ground plane, and provide approximately 55% open space to allow solar irradiance into the solar panel.

## 1. Introduction

Remote sensing is the technology of achieving information regarding climate change by a sensing device mounted on aircraft or spacecraft platform [1]. Nanosatellite technology can facilitate remote sensing applications including greenhouse gas monitoring, maritime tracking and messaging, multispectral Earth imaging etc. for academic, government, and commercial organizations [2]. Nanosatellites experience “smaller, cheaper, faster, better space missions” and introduce a revolutionary concept to remote sensing space research [3]. This concept has led to enthusiastic scientific, private, and government missions because of the use of low-cost miniature electronics with low power consumption [3,4]. Over the last few years, the number of nanosatellite missions has increased dramatically for low-earth orbit missions. Recently, nanosatellites have attracted attention for NASA space exploration missions beyond Earth’s orbit [5]. Every nanosatellite has some common functions for satellite operations, such as altitude control and determination, a power system, and uplink-downlink communications. Solar panels are used to manage the required power, and antennas are used to establish uplink-downlink communications. The ultra-high frequency (UHF)-band, especially the frequency range 420–450 MHz, is highly desirable for nanosatellite researchers, because the International Telecommunication Union has allocated this band as the International amateur satellite frequency band [6]. Antenna design for nanosatellites especially for lower frequencies presents its own challenge and has been a critical matter for CubeSat researchers [7]. In addition, solar panel integration presents another major problem because of the limited surface of the nanosatellite body [8]. These limitations were overcome by using deployable solar panels and antennas in recent nanosatellite missions [9]. However, mechanical deployment is quite sophisticated and this might increase the chance of mission failure [10]. Several small satellite missions have failed as a result of antenna deployment complexity [11,12] and solar panel deployment complexity [13]. In contrast to the deployable antenna, the patch antenna is an effective replacement to improve mission reliability. However, a UHF patch antenna occupies a large area of the nanosatellite body surface and introduces complexity in terms of integrating sufficient solar cells.

Various approaches have been reported to address the problem of integrating a solar panel with the antenna [14,15]. For instance, transparent antennas were placed above the solar panels on the CubeSat. Even though transparent antennas provide up to 90% of transparency for solar penetration, these types of antennas require expensive transparent substrates such as quartz or Lexan. In addition, their fabrication process is complex and expensive. Nontransparent subsolar antennas are placed beneath the solar panels with the constraint that the patch must not be covered by the solar panel, and this enlarges the antenna size for lower UHF applications [14,16]. Thus, designing an UHF antenna that is strategically integrated with the solar panel and that does not require mechanical deployment has become a major challenge for nanosatellite and antenna researchers.

This paper proposes a nanosatellite body-mounted slotted planner inverted F antenna (PIFA) antenna for a remote sensing UHF communication system. The antenna is designed to fit into commercially available 1U and 2U nanosatellites to avoid deployment complexity and to mitigate power scarcity. As the antenna structure is stable and, unlike the conventional deployable antenna, it does not require deployment, this antenna addresses the risk of mission failure due to antenna deployment. Moreover, as the proposed antenna provides space for additional placement of a solar panel, it helps to address the power scarcity in a nanosatellite. The proposed approach is a viable alternative based on the above-mentioned circumstances.

## 2. Materials and Methods

The proposed antenna is designed for established 1U, 2U and other nanosatellite structures, and addresses the problems associated with the limited volume and surface area for solar panel and antenna placement. First, the resonant condition for the nanosatellite structure was examined. Then an inverted-F antenna was designed, considering one face of the nanosatellite body as the ground plane that functions as an infinite ground plane to achieve lower UHF frequency. Sufficient space for placing the solar panel between the radiating element and ground plane was given priority in the initial design. The resonant frequency of the inverted-F antenna was initially estimated by the Equation (1) [17], where, *c* is the light velocity, and *L* and *W* are the length and width of the radiator, respectively.

(1) $$f=\frac{c}{4(L+W)}$$

**Figure 1:**
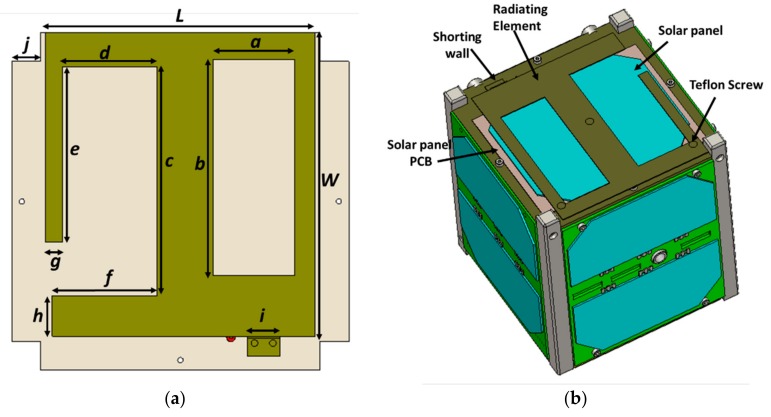
(**a**) Design layout of the proposed antenna; yellow radiating patch and gray ground plane, (**b**) antenna integrated with 1U nanosatellite structure, solar panel placed in between the radiating patch and the ground plane.

The design was optimized with the nanosatellite structure maintaining both electromagnetic and structural constraints. Estimation of the antenna performance was carried out with the commercially available numerical simulator Computer Simulation Technology (CST). The proposed lower UHF antenna is illustrated in Figure 1. The antenna consists of a rectangular radiating patch with a shorting wall, two linear slots on the patch, and a 50 Ω radio frequency (RF) coaxial feed. Two rectangular slots were etched out such that the equivalent inductance caused by the shorting pins and feeding probe were reduced, and more than fifty percent of the surface area of the radiating element remained uncovered to ensure proper solar penetration on the solar panel.

**Figure 2:**
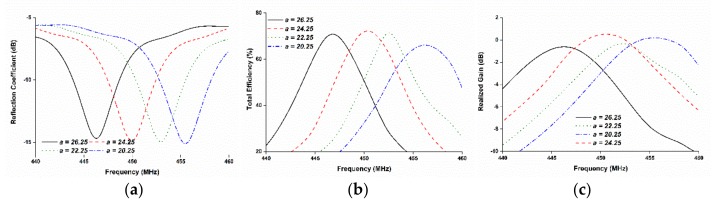
Parametric study on slot length *a*; (**a**) reflection coefficient, (**b**) total efficiency and, (**c**) realized gain: decreasing the slot length causes the resonant frequency to shift upwards.

The design parameters are distinctively studied to investigate their influences on the resonant frequency, and the other parameters remained unchanged. The effect of the rectangular slot width *a* on the antenna reflection coefficient, efficiency, and realized gain was studied, and is shown in Figure 2. The figure shows that decreasing the slot width causes the resonant frequency to shift upwards, i.e., an inverse relation is formed between the resonant frequency and slot width due to the change in effecting the electrical area. The total efficiency and realized gain were observed to have shifted in accordance with the resonant frequency.

**Figure 3:**
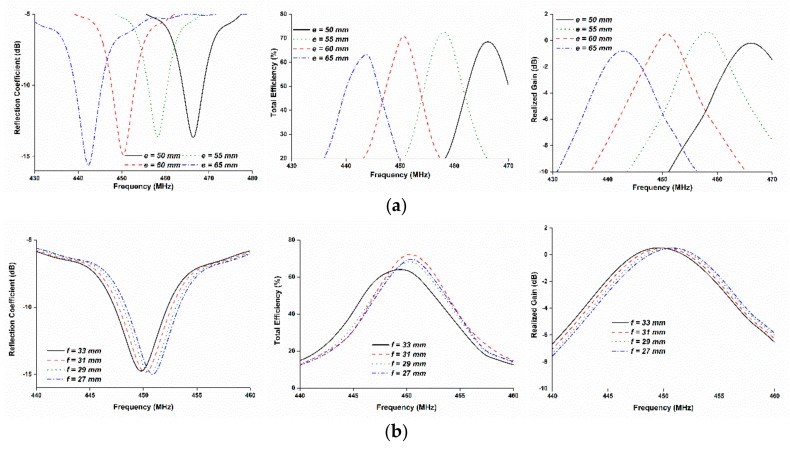
(**a**) Parametric study on the length of *e* and (**b**) parametric study on the length of the *f*: the radiating element *e* has a strong influence on the resonant frequency rather than *f*.

The dimension of the parameters *e* and *f* have an influence on the antenna reflection coefficient, total efficiency, and realized gain, as shown in Figure 3. It is observed from Figure 3 that, the radiating element *e* had a strong influence on the resonant frequency rather than *f*. Thus, it was easier to tune the resonant frequency by varying the length of *e*. It is seen from Figure 1 that variation of the slots dimension had a direct effect on the open space for solar penetration. Therefore, these parameters were numerically investigated.

**Figure 4:**
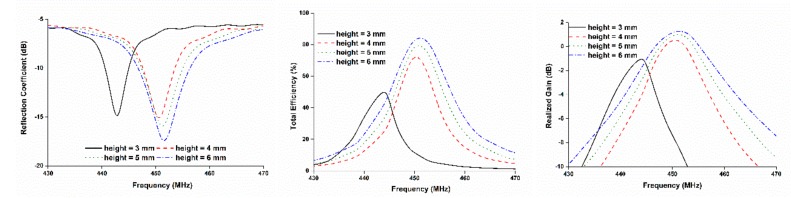
Parametric study of the gap between patch and ground plane; the gap cannot be increased randomly because of the design constraint of the satellite structure.

The effect of antenna performance on the gap between the radiating element and ground plane was investigated and is plotted in Figure 4. The resonating frequency remained almost unchanged as the gap height increased, but the gain and total efficiency increased. Despite the relation between the gap and antenna radiation performance, the gap between the radiating element and ground plane could not be increased randomly because of the constraint introduced by the satellite structure. On the other hand, when the gap was decreased to below 4 mm, the resonant frequency was shifted as well, thereby decreasing the gain and efficiency. This occurs because of the close interaction of the antenna radiator with the lossy solar panel [8]. Based on the aforementioned parametric study, the optimized antenna design parameters were arranged to achieve proper impedance matching, as tabulated in Table 1.

**Figure 5:**
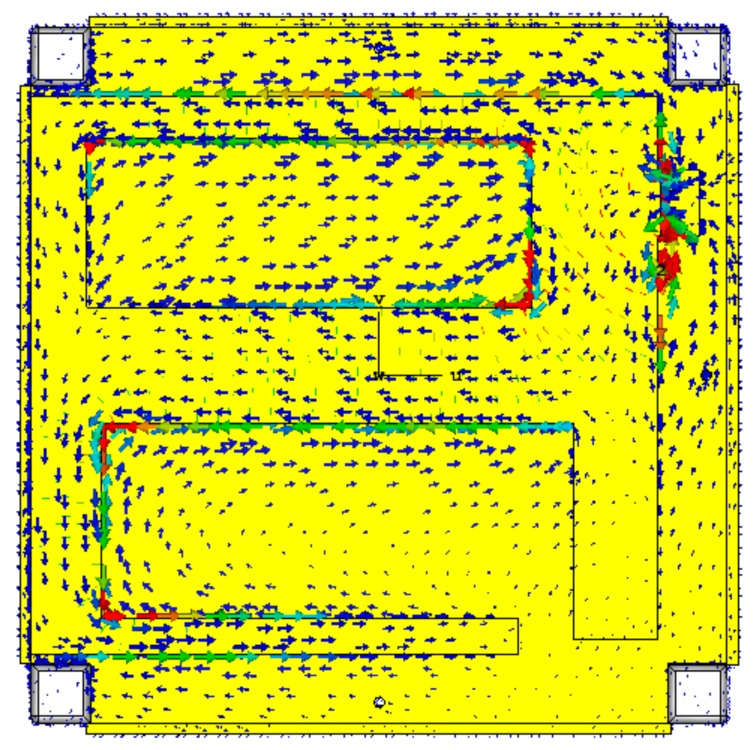
Surface current distribution of the planner inverted F antenna (PIFA) antenna at 450 MHz; starting from the feed point, the travelling current forms a larger electrical path to achieve resonance at a lower frequency.

The surface current distribution of the modified PIFA is depicted in Figure 5. The majority current was observed in the vicinity of the shorting wall and some strong current arose at the slot corner. This indicates the formation of a larger electrical patch to achieve lower frequency, even though more than 55% of the radiating element was truncated. Moreover, the current flow was distributed across the structure as well as the ground plane, which reduced interference with external objects that affected antenna performance.

## 3. Results

**Figure 6:**
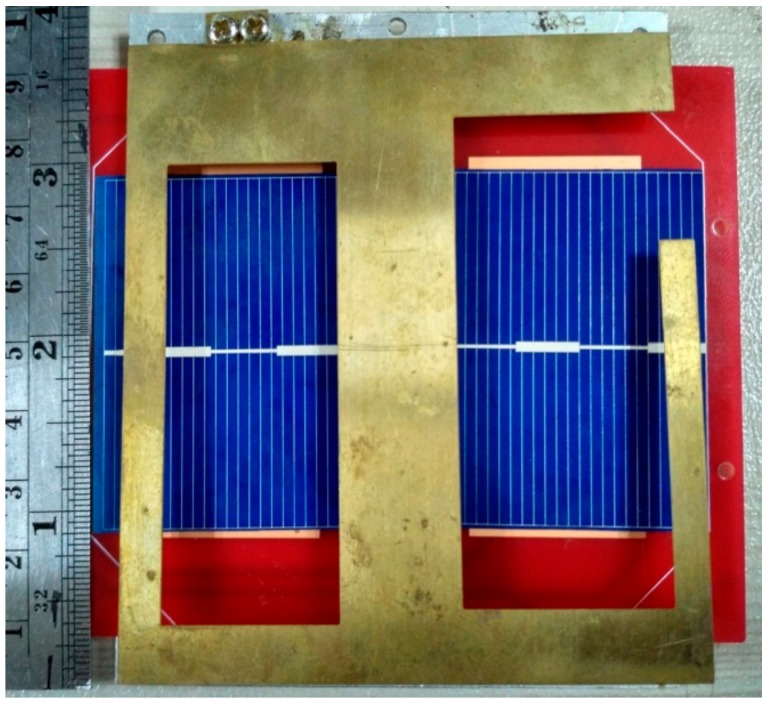
Fabricated prototype of the proposed antenna integrated with the solar panel; the solar panel is placed in between the radiating patch and ground plane.

**Figure 7:**
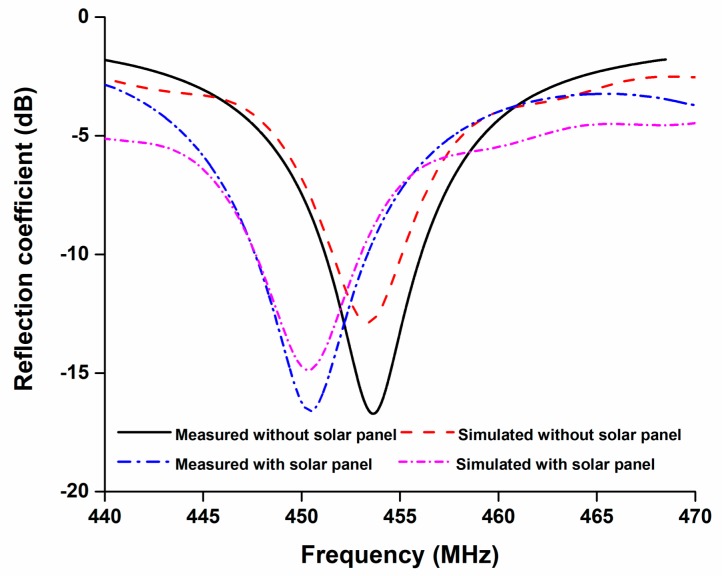
Simulated and measured reflection coefficient of the proposed antenna. Reflection coefficients are measured with and without the solar panel and the measured −10 dB operating bandwidth is 447.5 MHz–453.5 MHz.

The antenna was fabricated according to the optimized parameters listed in Table 1, illustrated in Figure 6. The reflection coefficient of the antenna was analyzed in two conditions, one was without integrating solar panel and another was integrating solar panel between antenna and ground plane, shown in Figure 7. The solar panel affected the antenna impedance of the antenna and shifted the resonant frequency by 3 MHz. The resonant frequency was tuned by optimizing its parameters. The proposed antenna achieved a −10 dB impedance bandwidth of 6.0 MHz (447.5 MHz–453.5 MHz).

**Figure 8:**
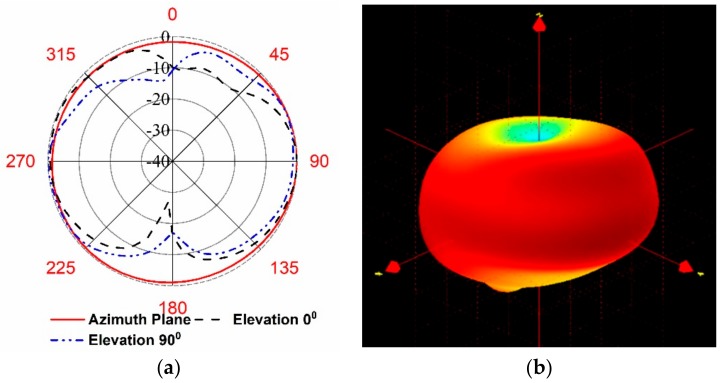
Measured radiation pattern at 450 MHz (**a**) azimuth and elevation (**b**) 3D pattern showing omnidirectional radiation characteristics.

**Figure 9:**
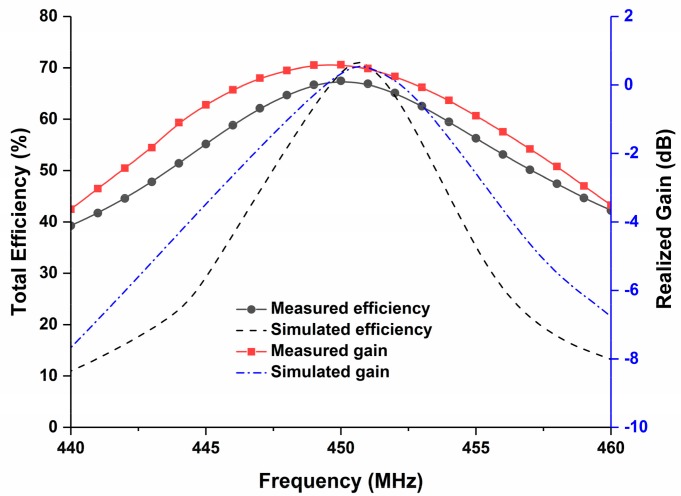
Simulated and measured total efficiency and gain of the fabricated prototype; where 67.45% efficiency is achieved with 0.6 dB of realized gain.

The radiation characteristics of the proposed UHF antenna with the satellite structure were measured in the Satimo near-field measurement system. The measured radiation pattern in both the azimuth and elevation planes, along with the 3D pattern at 450 MHz is illustrated in Figure 8, where it can be observed that the antenna shows a nearly omnidirectional radiation pattern at the azimuth plane. According to the results in Figure 9, the total efficiency of the proposed UHF antenna at the resonance frequency was approximately 67.45% with 0.6 dB of realized gain, although the lossy nature of the solar cells degraded the antenna efficiency.

**Figure 10:**
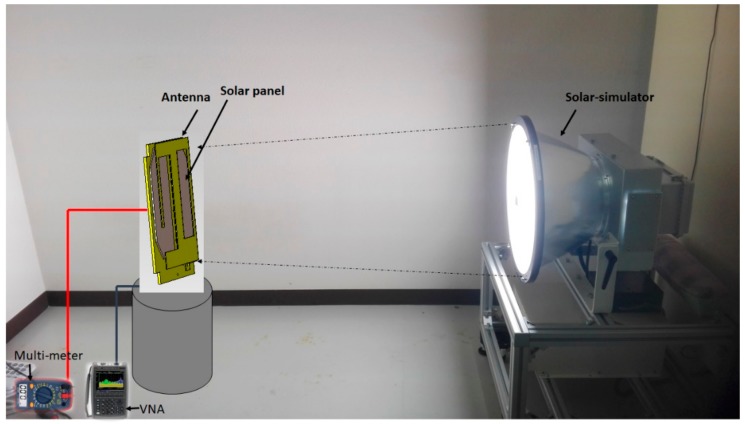
Solar panel output power measurement setup. The components are: Solar-simulator, vector network analyzer (VNA), Multi-meter and the proposed Antenna. The output power measurement was conducted using a fixed load.

**Figure 11:**
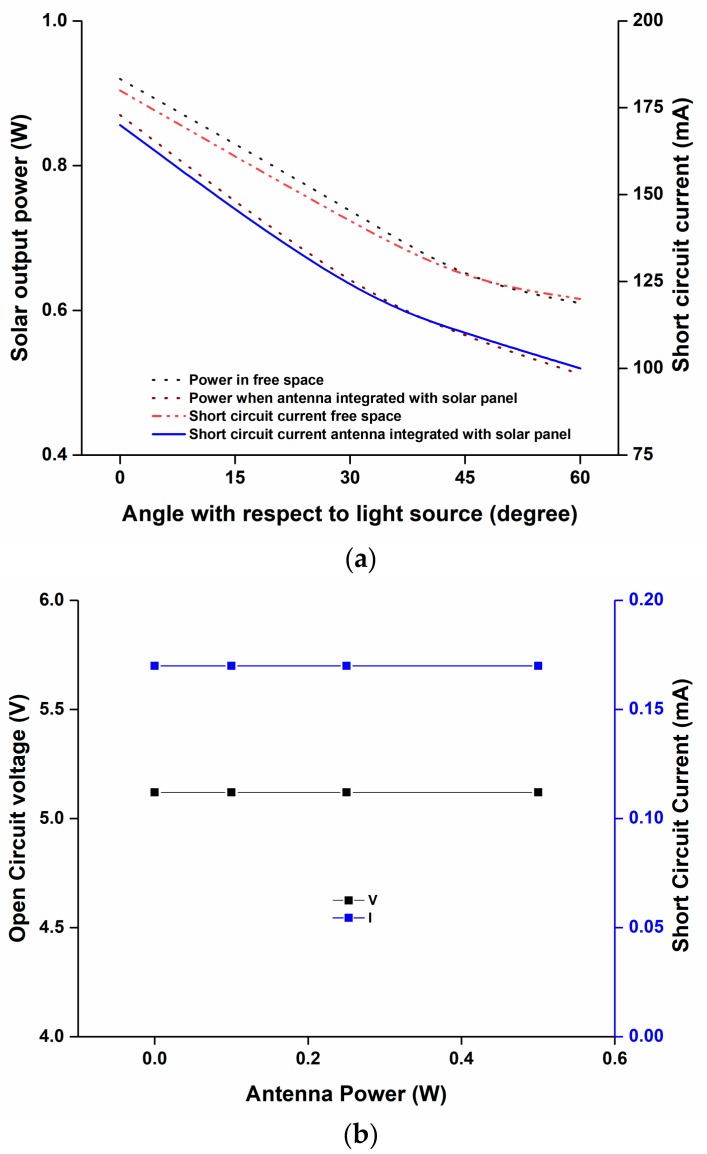
(**a**) Solar output power and short circuit current measurement without and with antenna placement; (**b**) Solar characterization with varying antenna power from 0.1 W to 0.5 W.

The output power of the solar panel was measured using the solar simulator. The schematic layout of the measurement setup is depicted in Figure 10. At first, the solar panel was placed in the setup and the output power was measured. The output power measurement was conducted using a fixed load. Then the solar panel was placed between the antenna and the ground plane and the solar panel output power was measured. Both results are plotted in Figure 11a, which shows that more than 75% of the solar output was achieved compared with the open space solar output condition. The respective short-circuit current was also shown in the figure. Moreover, according to [18] the open circuit voltage and short circuit current had the open circuit voltage and the short circuit current was investigated by varying the antenna input power from 0.1 W to 0.5 W; this is plotted in Figure 11b. This investigation confirms that the antenna radiation power had no visible effect on the solar panel performance.

**Figure 12:**
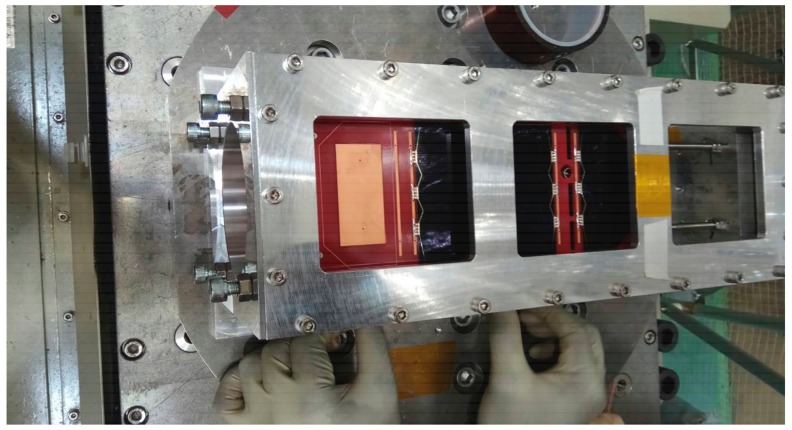
Vibration test setup of the proposed antenna with nanosatellite structure. Aluminum 7075 material based 2U nanosatellite structure was considered for the test as 100+/−0.1 mm wide (+X, +Y axes); 227+/−0.1 mm tall (+Z axis) and maximum weight was 2.6 kg.

Random vibration and sine vibration tests of the proposed antenna integrated with nanosatellite structure were carried out to examine the stability of the antenna structure under vibration conditions. The test was performed in the Laboratory of Spacecraft Environment Interaction Engineering (LaSEINE), Kyushu Institute of Technology, Japan. The test setup is presented in Figure 12. No malfunctions occurred during excitation and after excitation. The antenna was in the exact position as it was before the vibration test. A summary of the antenna is presented in Table 2. The performance of the proposed antenna was compared with existing UHF antennas, tabulated in Table 3. Considering the comparison criteria in the lower UHF band, it can be seen that the proposed antenna was a potential candidate for the nanosatellite communication system.

The proposed design provides a significant trade-off between the antenna size and performance in terms of gain, efficiency and operating band. In context of deployment, nanosatellite engineers have to go through complexities for the design and operation of the deployment mechanism. Certainly, the proposed antenna has the potential to provide deployment-free operation and this allows the satellite engineers to focus on other design criteria. The amount of gain provided by the antenna is satisfactory for smooth uplink and downlink communication in the UHF band. Moreover, the gain achieved by the antenna is measured while antenna is integrated with the nanosatellite structure. So, there is no risk of decreasing the gain after integration with the nanosatellite structure that usually happens in patch antennas. There are commercially available UHF nanosatellite antennas that have approximately 0 dB gain [26]. Based on the aforementioned design constraints, the proposed antenna has achieved its potential for nanosatellite UHF remote sensing applications. 

## 4. Conclusions

This communication presents the modified PIFA antenna, which was designed to exclusively feature a UHF application with 55% of open space on a remote sensing nanosatellite’s single face for solar irradiance penetration. The antenna was fabricated and integrated with a 1U nanosatellite structure. The antenna shows S11 < −15 dB at 450 MHz with 67.45% total efficiency and 0.6 dB realized gain. This proposed antenna will facilitate the sensing nanosatellite’s power scarcity.

## Figures and Tables

**Table 1:** Antenna design parameters.

Parameters	Value (mm)	Parameters	Value (mm)
*L*	80	*e*	60
*W*	90	*f*	31
*a*	24.25	*g*	5
*b*	64	*h*	12
*c*	68	*i*	9.6
*d*	28	*j*	8.5

**Table 2:** Summary of the proposed ultra-high frequency (UHF) antenna.

Parameter	Specifications	Unit
Frequency	447.5–453.5	MHz
Impedance bandwidth (−10 dB)	6	MHz
Polarization	Linear	-
Realized Gain	0.6	dB
Antenna Size	80 × 90	mm
Efficiency	67.45	%
Material	Brass	-
RF input	50 Ω mmcx connector	-
Vibration Test	Qualified	-

RF: radio frequency.

**Table 3:** Comparison of Characteristics of Different UHF antennas.

Ref. No.	Antenna Type	Operating Frequency (MHz)	Antenna Size (mm)	Gain	Solar Integration Facility	Remarks
[19]	Printed patch	410–485	220 × 220 × 28.5	5.2 dB	no	Too large for 1U, 2U nanosatellites.
[20]	Microstrip patch	435–437	170 × 120 × 6.4	0.7 dB	no	Too large to fit with 1U nanosatellites.
[21]	Fractal shaped	700–4710	120 × 120 × 1.6	1.71 dB	no	Not compact enough. Does not cover lower UHF frequency (450 MHZ)
[14]	Printed patch	427.38–437.17	320 × 80 × 3.17	2.12 dB	no	Large dimension
[22]	Microstrip patch	384–410	150 × 150 × 37	0.4 dB	no	Low gain with large dimension.
[23]	Dipole	430	160	2.15 dB	yes	Externally mounted with satellite structure
[24]	Monopole	435–438	175	2.35 dBi	yes	Deployable complexity
[25]	Monopole	146 & 438	513.6 × 10 × 10	2.06 dBi at 146 MHz 3.35 dBi at 438 MHz	yes	Deployable complexity
Proposed Antenna	Modified PIFA	447.5–453.5	80 × 90 × 0.5	0.6 dB	yes	Deployable complexity free
